# Resistant Hypertension, Patient Characteristics, and Risk of Stroke

**DOI:** 10.1371/journal.pone.0104362

**Published:** 2014-08-04

**Authors:** Chen-Ying Hung, Kuo-Yang Wang, Tsu-Juey Wu, Yu-Cheng Hsieh, Jin-Long Huang, El-Wui Loh, Ching-Heng Lin

**Affiliations:** 1 Cardiovascular Center, Taichung Veterans General Hospital, Taichung, Taiwan; 2 Department of Internal Medicine, Taipei Veterans General Hospital, Hsinchu Branch, Hsinchu County, Taiwan; 3 School of Medicine, Chung Shan Medical University, Taichung, Taiwan; 4 Department of Internal Medicine, Faculty of Medicine, Institute of Clinical Medicine, Cardiovascular Research Center, National Yang-Ming University School of Medicine, Taipei, Taiwan; 5 Kaohsiung Municipal Kai-Syuan Psychiatric Hospital, Kaohsiung, Taiwan; 6 Department of Medical Research, Taichung Veterans General Hospital, Taichung, Taiwan; Shanghai Institute of Hypertension, China

## Abstract

**Background:**

Little is known about the prognosis of resistant hypertension (RH) in Asian population. This study aimed to evaluate the impacts of RH in Taiwanese patients with hypertension, and to ascertain whether patient characteristics influence the association of RH with adverse outcomes.

**Methods and Results:**

Patients aged ≥45 years with hypertension were identified from the National Health Insurance Research Database. Medical records of 111,986 patients were reviewed in this study, and 16,402 (14.6%) patients were recognized as having RH (continuously concomitant use of ≥3 anti-hypertensive medications, including a diuretic, for ≥2 years). Risk of major adverse cardiovascular events (MACE, a composite of all-cause mortality, acute coronary syndrome, and stroke [included both fatal and nonfatal events]) in patients with RH and non-RH was analyzed. A total of 11,856 patients experienced MACE in the follow-up period (average 7.1±3.0 years). There was a higher proportion of females in the RH group, they were older than the non-RH (63.1 vs. 60.5 years) patients, and had a higher prevalence of cardiovascular co-morbidities. Overall, patients with RH had higher risks of MACE (adjusted HR 1.17; 95%CI 1.09–1.26; p<0.001). Significantly elevated risks of stroke (10,211 events; adjusted HR 1.17; 95%CI 1.08–1.27; p<0.001), especially ischemic stroke (6,235 events; adjusted HR 1.34; 95%CI 1.20–1.48; p<0.001), but not all-cause mortality (4,594 events; adjusted HR 1.06; 95%CI 0.95–1.19; p = 0.312) or acute coronary syndrome (2,145 events; adjusted HR 1.17; 95%CI 0.99–1.39; p = 0.070) were noted in patients with RH compared to those with non-RH. Subgroup analysis showed that RH increased the risks of stroke in female and elderly patients. However, no significant influence was noted in young or male patients.

**Conclusions:**

Patients with RH were associated with higher risks of MACE and stroke, especially ischemic stroke. The risks were greater in female and elderly patients than in male or young patients.

## Introduction

Hypertension is one of the most important cardiovascular problems and is associated with an increased risk of stroke, myocardial infarction, and mortality [1], [2]. It is also one of the most important modifiable risk factors for cardiovascular diseases [2]. Resistant hypertension (RH) represents a potentially higher risk subset of the disease and is associated with higher cardiovascular morbidity and mortality. A recent scientific statement from the American Heart Association and the European Society of Cardiology defined RH as uncontrolled blood pressure (BP) despite patient adherence to 3 anti-hypertensive drugs (including a diuretic), or controlled BP when using ≥4 anti-hypertensive drugs [3], [4].

Previous studies indicated that 12% to 30% of patients with hypertension in Western countries may have RH [3], [5], [6], but the exact prevalence of RH in Asian population has not been examined in advanced. The observation that many patients with hypertension have high BP despite the use of multiple anti-hypertensive drugs, has led to an increased interest in the independent role of RH [3]. A greater understanding of the prevalence and prognosis of RH is important to improve the management of these patients. Therefore, RH has been defined as a major current focus of hypertension research by the American Heart Association [3].

In this study, we investigated the prognosis of RH in an Asian population. The purpose of the present study was to evaluate the association of RH with major adverse cardiovascular events (MACE) in a large cohort of hypertensive patients in Taiwan. We compared the risk of all-cause mortality, acute coronary syndrome, and stroke between patients with RH and non-RH. We also wanted to determine if demographic data or cardiovascular co-morbidity could predict the influence of RH.

## Methods

### Research database

The National Health Insurance program, which was implemented in Taiwan in 1995, covers about 99% of the island's population. The National Health Research Institute (NHRI) has established the National Health Insurance Research Database. We used a systemic sampling of patient data, which was released by the NHRI (from 2000 to 2011 with a total of 1,000,000 subjects), for the current analysis. The development of the research database has been described in detail elsewhere [7], [8]. In brief, the random samples have been confirmed by the NHRI to be representative of the general population. The NHRI made data available at the individual level in an anonymous format, and safeguarded the privacy of individuals. This study was approved by the Institutional Review Board of Taichung Veterans General Hospital.

### Study population

Patients aged ≥45 years with hypertension were identified according to the International Classification of Diseases, Ninth Revision, Clinical Modification (ICD-9-CM) code 401–405. Since the lack of BP data in our research database, we use medication using information to avoid misclassifications and ensure the diagnostic validity. Only patients who had a diagnosis of hypertension and were treated with anti-hypertensive drugs in the period 2000–2010 were selected. Patients were not eligible for enrollment in this cohort study if they had a history of atrial fibrillation, atrial flutter, heart failure, stroke, or acute coronary syndrome. This dynamic cohort included 111,986 patients for analysis. According to the guidelines of the Taiwan Society of Cardiology for the management of hypertension, hypertension was defined as systolic BP ≥140 mmHg or diastolic BP ≥90 mmHg, with lower cutoffs of systolic BP ≥130 mmHg or diastolic BP ≥80 mmHg for high-risk patients, such as those with chronic kidney disease, diabetes, stroke, coronary artery disease or its equivalents [9].

### Definition of RH

Anti-hypertensive medication records were retrieved from ambulatory and inpatient claims data. Medications were identified based on drug class, such as angiotensin-converting enzyme inhibitors (ACEIs) or angiotensin receptor blockers (ARBs), alpha blockers, beta blockers, calcium channel blockers, diuretics, spironolactone, aspirin, clopidogrel, warfarin, statins, oral hypoglycemic agents, and insulins. Each medication in combination anti-hypertensive pills was counted as a separate class of drug. Patients were divided into a RH and a non-RH group according to their medication use in the period 2000–2010. RH was defined as a hypertension with continuously concomitant use of ≥3 anti-hypertensive medications, including a diuretic, for ≥2 years (at any time during 2001–2010). Hypertensive patients who did not meet our criteria for RH were classified into the non-RH group.

### Definition of Outcomes

The primary outcome of this study was defined as MACE, which was a composite of all-cause mortality, acute coronary syndrome, and stroke (included both fatal and nonfatal events), whichever occurred first. Other outcomes included all-cause mortality, acute coronary syndrome, overall stroke, ischemic stroke and hemorrhagic stroke. The study endpoint was defined as any events after patients being classified into RH or non-RH groups during the 11-year follow-up period (2001–2011). To ensure the diagnostic validity, only patients with at least 1 inpatient hospitalization diagnosis of acute coronary syndrome or stroke were identified. Other co-morbidities were identified by ICD-9-CM diagnostic code (at least 1 inpatient hospitalization diagnosis or at least 3 consensus diagnoses at an outpatient department) when inclusive: diabetes mellitus, hyperlipidemia, ischemic heart disease, peripheral vascular disease, valvular heart disease, pulmonary disease, and renal disease. Charlson co-morbidity index was also calculated for each patient.

### Statistical analysis

The data are presented as the mean values and standard deviations (SD) for continuous variables, and proportions for categorical variables. The differences between values were analyzed by using t test for continuous variables, and chi-square test for categorical variables. The MACE-free survival curves were plotted via the Kaplan–Meier method with statistical significance examined by the log-rank test. Multivariable Cox proportional hazards regression models were used to identify independent factors contributing to MACE occurrence (adjusted for age, sex, co-morbidities, Charlson co-morbidity index, and medications). For further controlling the potential confounding bias, we performed multivariable analyses by using a Cox proportional hazards regression model that adjusting for age, sex, co-morbidities, Charlson co-morbidity index, and medications. All statistical analyses were carried out by SAS software version 9.2 (SAS Institute, Inc., Cary, NC, USA). A p value of <0.05 was considered statistically significant.

## Results

### Clinical characteristics

A total of 111,986 hypertensive patients aged ≥45 years were enrolled in this study, of whom 16,402 (14.6%) patients met our definition of RH. All patients were followed up for a maximum period of 11 years (average 7.1±3.0 years). The mean age of the study population was 60.9±10.6 years, with 34.5% of them aged ≥65 years. Females accounted for 49.6% of the population. The average Charlson co-morbidity index of the cohort was 1.1±1.5. Among patients with RH, 36.8% were on 4 anti-hypertensive agents, and 7.4% on ≥5 agents. Anti-hypertensive drugs used by patients with RH included ACEIs or ARBs (82.6%), alpha blockers (15.6%), beta blockers (62.1%), calcium channel blockers (87.4%), and spironolactone (4.4%); by definition, all patients with RH used diuretics.

Table 1 summarizes the baseline characteristics of the patients with RH and non-RH. Patients with RH were older (63.1 vs. 60.5 years; p<0.001), more likely to be female (50.9% vs. 49.4%; p<0.001), and had a higher prevalence of co-morbidities, such as diabetes mellitus, hyperlipidemia, ischemic heart disease, peripheral vascular disease, valvular heart disease, pulmonary disease, and renal disease, when compared to those with non-RH (p values <0.001). The RH group had higher rates of drug use, including anti-hypertensives, aspirin, clopidogrel, warfarin, statins, oral hypoglycemic agents, and insulins than the non-RH group (p values <0.001). Patients with RH also has a higher Charlson co-morbidity index than those with non-RH (1.4 vs.1.0; p<0.001).

**Table 1:** Baseline characteristics.

	Original cohort (n = 111,986)		
	RH	Non-RH	*P* value
	(n = 16,402, 14.6%)	(n = 95,584, 85.4%)	
Variables	No. (%)	No. (%)	
**Age at entry, years**			
mean ± SD	63.1±10.5	60.5±10.5	<0.001
45–54	4,610 (28.1)	36,126 (37.8)	<0.001
55–64	4,604 (28.1)	27,938 (29.2)	
65–74	4,743 (28.9)	21,284 (22.3)	
≧75	2,445 (14.9)	10,236 (10.7)	
**Female**	8,344 (50.9)	47,167 (49.4)	<0.001
**Co-morbidities**			
Diabetes mellitus	4,686 (28.6)	14,851 (15.5)	<0.001
Hyperlipidemia	3,656 (22.3)	11,495 (12.0)	<0.001
Ischemic heart disease	3,288 (20.1)	9,102 (9.5)	<0.001
Peripheral vascular disease	333 (2.0)	1,012 (1.1)	<0.001
Valvular heart disease	619 (3.8)	1,637 (1.7)	<0.001
Pulmonary disease	1,729 (10.5)	7,573 (7.9)	<0.001
Renal disease	718 (4.4)	2,176 (2.3)	<0.001
**Charlson co-morbidity index**			
mean ± SD	1.4±1.6	1.0±1.5	<0.001
**Medications**			
ACEIs or ARBs	13,555 (82.6)	40,633 (42.5)	<0.001
Alpha blockers	2,552 (15.6)	5,459 (5.7)	<0.001
Beta blockers	10,180 (62.1)	32,601 (34.1)	<0.001
Calcium channel blockers	14,337 (87.4)	43,896 (45.9)	<0.001
Diuretics	16,402 (100.0)	16,999 (17.8)	<0.001
Spironolactone	723 (4.4)	934 (1.0)	<0.001
Aspirin	5,224 (31.9)	10,879 (11.4)	<0.001
Clopidogrel	333 (2.0)	957 (1.0)	<0.001
Warfarin	138 (0.8)	499 (0.5)	<0.001
Statins	2,901 (17.7)	5,459 (5.7)	<0.001
Oral hypoglycemic agents	4,176 (25.5)	8,169 (8.6)	<0.001
Insulins	887 (5.4)	1,125 (1.2)	<0.001

ACEI = angiotensin-converting enzyme inhibitor; ARB = angiotensin receptor blocker; RH = resistant hypertension; SD = standard deviations.

### Outcomes

**Figure 1:**
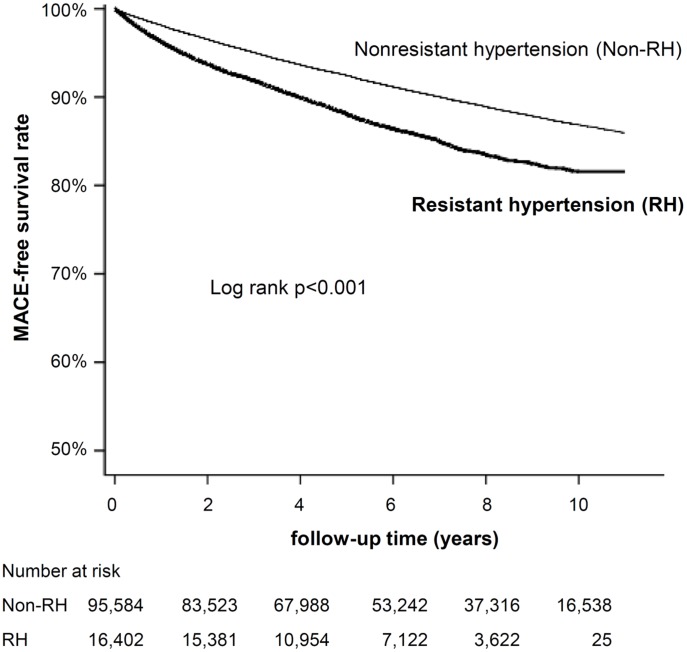
Major adverse cardiovascular events (MACE)-free survival rate between resistant hypertension (RH) and non-RH groups.

The Kaplan-Meier survival plot presented in Figure 1 shows the MACE-free survival rate between RH and non-RH group. The non-RH group had a higher survival probability than the RH group (log rank p<0.001). Table 2 demonstrates the adjusted hazard ratio (HR) for the development of MACE in the cohort. During the follow-up period, 11,856 patients (10.6% of the study population) developed MACE. MACE occurred more frequently in RH group when compared with non-RH group before and after adjustment (13.9 vs. 10.0%; adjusted HR 1.17; 95% confidence interval [CI] 1.09–1.26; p<0.001). Patients with RH had a 17% higher risk of the primary endpoint (all-cause mortality, acute coronary syndrome, or stroke) when compared to patients with non-RH. The rise was likely to be caused by frequent stroke events in RH group (10,211 events; 11.8 vs. 8.7%; adjusted HR 1.17; 95% CI 1.08–1.27; p<0.001), especially ischemic stroke (6,235 events; 7.4 vs. 5.3%; adjusted HR 1.34; 95% CI 1.20–1.48; p<0.001). Rates of all-cause mortality (4,594 events; adjusted HR 1.06; 95% CI 0.95–1.19; p = 0.312), acute coronary syndrome (2,145 events; adjusted HR 1.17; 95% CI 0.99–1.39; p = 0.070), and hemorrhagic stroke (1,967 events; adjusted HR 0.96; 95% CI 0.80–1.15; p = 0.634) were similar.

**Table 2:** Adjusted HR for MACE.

	Model 1: original cohort (n = 111,986)***				Model 2 (n = 105,277)****	
	RH	Non-RH	Adjusted HR (95% CI)**	*P* value	Adjusted HR (95% CI)**	*P* value
Outcomes	No. (%)	No. (%)				
**MACE**	2,283 (13.9)	9,573 (10.0)	1.17 (1.09–1.26)	<0.001	1.12 (1.05–1.19)	0.001
**All-cause mortality**	882 (5.4)	3,712 (3.9)	1.06 (0.95–1.19)	0.312	1.05 (0.95–1.17)	0.299
**Acute coronary syndrome** *	459 (2.8)	1,686 (1.8)	1.17 (0.99–1.39)	0.070	1.16 (1.00–1.35)	0.047
**Stroke** *	1,933 (11.8)	8,278 (8.7)	1.17 (1.08–1.27)	<0.001	1.11 (1.03–1.19)	0.004
**Ischemic stroke** *	1,209 (7.4)	5,026 (5.3)	1.34 (1.20–1.48)	<0.001	1.24 (1.14–1.36)	<0.001
**Hemorrhagic stroke** *	345 (2.1)	1,622 (1.7)	0.96 (0.80–1.15)	0.634	0.98 (0.83–1.15)	0.794
**Unclassified stroke** *	379 (2.3)	1,630 (1.7)	0.96 (0.80–1.14)	0.625	1.00 (0.86–1.16)	0.976

* Included both fatal and nonfatal events.

** Adjusted for age, sex, co-morbidities, Charlson co-morbidity index, and medications.

*** Model 1: original cohort recruited patients with diagnosis of hypertension (ICD-9-CM code 401–405) for analysis.

**** Model 2: recruited only patients with diagnosis of essential hypertension (ICD-9-CM code 401) for analysis.

CI = confidence interval; HR = hazard ratio; MACE = major adverse cardiac events; RH = resistant hypertension.

### Sensitivity analysis

When a strict definition (patients on ≥4 agents, including a diuretic) was used for RH, the overall prevalence of the disease decreased from 14.6% to 6.5% (Table not shown). The strictly defined RH was still associated with a significant hazard for the primary endpoint in these patients on multivariate adjusted analyses (adjusted HR 1.26; 95% CI 1.15–1.39; p<0.001) when compared to pure non-RH group (patients on <3 agents or without a diuretic). There was a significant association between RH and all-cause mortality (adjusted HR 1.21; 95% CI 1.04–1.41; p = 0.012), and between RH and acute coronary syndrome (adjusted HR 1.35; 95% CI 1.09–1.68; p = 0.007); all other outcomes were similar to the primary definition (For stroke: adjusted HR 1.24; 95% CI 1.11–1.37; p<0.001. For ischemic and hemorrhagic stroke, the adjusted HR were 1.40 [95% CI 1.22–1.61; p<0.001] and 1.13 [95% CI 0.89–1.42; p = 0.313]).

When we recruited only those with diagnosis of essential hypertension (ICD-9-CM code 401), a total of 105,277 patients were enrolled in analysis. The overall prevalence of RH was similar (13.8%) to the original model. The new model revealed that RH was still associated with a significant hazard for the primary endpoint in these patients on multivariate adjusted analyses (adjusted HR 1.12; 95% CI 1.05–1.19; p = 0.001) when compared to non-RH group (see model 2 of table 2). There was a significant association between RH and acute coronary syndrome (adjusted HR 1.16; 95% CI 1.00–1.35; p = 0.047), and other outcomes were similar to the primary definition (For stroke: adjusted HR 1.11; 95% CI 1.03–1.19; p = 0.004. For ischemic and hemorrhagic stroke, the adjusted HR were 1.24 [95% CI 1.14–1.36; p<0.001] and 0.98 [95% CI 0.83–1.15; p = 0.794]).

### Subgroup analysis

**Figure 2:**
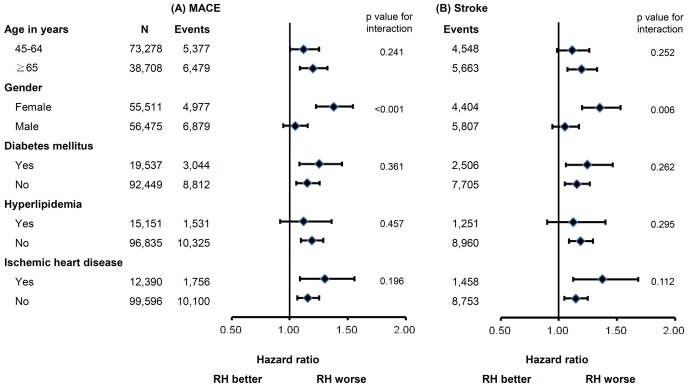
Subgroup analysis for (A) major adverse cardiovascular events (MACE), and (B) stroke.

Figure 2 displays the subgroup analysis for MACE and stroke occurrence based on Cox proportional hazards analysis with RH as a covariate. The relationships of baseline characteristics and co-morbidities and the risk of MACE and stroke were evaluated in patients with RH and with non-RH. Female (adjusted HR 1.38; 95% CI 1.23–1.55; p<0.001) and elderly (adjusted HR 1.20; 95% CI 1.09–1.33; p<0.001) RH patients had significantly worse outcome than non-RH patients. A borderline significance was noted in developing MACE in younger patients (aged 45–64 years; adjusted HR 1.12; 95% CI 1.00–1.26; p = 0.048). However, no significant elevated risk was found in male patients (adjusted HR 1.05; 95% CI 0.95–1.15; p = 0.375). No significant difference in developing stroke was found between RH and non-RH groups in male (adjusted HR 1.05; 95% CI 0.95–1.17; p = 0.337) and younger patients (adjusted HR 1.12; 95% CI 0.99–1.26; p = 0.080). On the other hand, there was a significant difference between RH and non-RH groups in female (adjusted HR 1.35; 95% CI 1.20–1.53; p<0.001) and elderly (adjusted HR 1.20; 95% CI 1.08–1.33; p = 0.001). Meanwhile, patients with and without any cardiovascular co-morbidities had similar hazards of these outcomes (HR ranged from 1.12 to 1.38).

## Discussion

### Main findings

This nationwide cohort study is one of the largest studies (enrolled 111,986 subjects) with the longest follow-up period (from 2001 to 2011) for analysis the prognosis of RH patients in Asian population. The main results of this study were that the risk of MACE was higher in RH patients than non-RH patients, especially for female and elderly patients. RH was associated with a significant increase in the risk of MACE and stroke, especially ischemic stroke. Our study also showed that female gender and old age predicts the influence of RH. Female patients with RH were 35% more likely to experience a stroke event when comparing to those with non-RH, while no such difference was noted in male patients.

### The association of RH with MACE

Our study found that RH was associated with long-term MACE, especially stroke, independent of other factors known to influence the long-term outcomes. An USA registry, which followed 205,750 patients for 3.8 years, found that RH was associated with a higher risk of cardiovascular events [6]. The international Reduction of Atherothrombosis for Continued Health (REACH) registry followed 53,530 hypertensive patients for 4 years found an 11% increased risk of the adverse long-term outcomes (a composite of cardiovascular death, myocardial infarction, or stroke), especially the non-fatal stroke risk [10]. Our study showed similar results and is consistent with current understanding on stroke risk and elevated BP [11]. This finding is especially critical in Asian population since few studies have directly compared cardiovascular outcomes between RH and non-RH patients in this population. Furthermore, the increase in cardiovascular events in this cohort was probably due to a 34% increase in the risk of ischemic stroke.

### Age, Gender, and MACE

Herein, we demonstrated that age and gender were convenient and useful characteristics for predicting the influence of RH. Female and elderly patients were associated with a 35% and 20% increased risk of stroke, respectively. On the other hand, male and young patients had no significantly increased stroke risk from RH. This implies that the gender and age can be used to predict the cardiovascular risk in patients with RH. Although male with hypertension in general had a higher cardiovascular risk than female [12]–[14], limited information was available regarding the impact of RH on male and female separately. In a recent analysis of the Women's Ischemia Syndrome Evaluation (WISE) study [15], female with RH had a greater long-term risk of adverse events when compared to female with non-RH. This result partially supports our findings: RH was associated with increase risks of MACE in female.

Male and female had different clinical characteristics and diverse results of treatment in the aspect of resistant hypertension. There was a higher proportion of female in RH populations than non-RH populations in our cohort as well as other studies [10], [16]. Female is more likely to be prescribed antihypertensive medications, but has a lower rate of BP control than male, especially in the elderly [16]–[19]. Our study further revealed that female with RH was associated with a profoundly increased risk of stroke compared with female with non-RH. Some studies may support these findings. Tang et al indicated a heterogeneous contribution of risk factors for stroke between male and female in a Chinese cohort [20]; obesity and hypertension were risk factors for stroke in female, whereas dyslipidemia were associated with stroke in male. Another study conducted by Kim et al further supported the findings that risk factors for cerebral atherosclerosis differ between genders. Hypertension was the most important risk factor for females to develop cerebral atherosclerosis, while diabetes and hypercholesterolemia for males [21]. While this is the first study to explore the relationship between gender, age, and MACE in patients with RH, further research is needed to confirm this relationship and to identify the exact mechanisms involved.

### Prevalence of RH in Taiwan

A recent report suggests that the prevalence of hypertension was around 10.5% to 13.3%, with only slightly increased in recent years, in a Southern Chinese population [22]. Meanwhile, current RH prevalence estimates vary between studies. Data from large clinical trials suggest that a third of hypertensive patients were on ≥3 BP controlling agents [3], [5]. The estimated prevalence of RH was around 8.9% in the hypertensive population in the period 2003–2008 in a recent analysis of the USA National Health and Nutrition Examination Survey data [23], and RH became 20.7% in the period 2005–2008. A Spanish registry [24] and a USA cross-sectional study [25] found around 12% of hypertensive patients met the criteria for RH. In Asian, the Japanese J-HOME study reported a prevalence of RH of 13% [26]. Our cohort showed a similar finding that 14.6% of the hypertensive patients in Taiwan met the definition of RH.

### Strength and limitations

The major strength of this study is that the research subjects were sampled from a large community cohort. Importantly, we showed that the risk of MACE was significantly higher in those with RH than with non-RH in an Asian population. Several limitations should be considered when interpreting the present study. First, the study population included mainly Taiwanese people, and we did not have the details of ethnic data for further analysis. Second, information regarding levels of BP and duration of hypertension were not available in the research database. Therefore, we cannot clarify the BP levels or the BP control rate of RH and non-RH groups. In order to address this limitation, we conducted sensitivity analyses by varying the definition of RH. Using a strict definition (patients on ≥4 agents) or recruiting only patients with essential hypertension resulted in similar HRs for the primary endpoint. Third, anti-hypertensive drug use was defined at baseline when these patients were divided into RH or non-RH groups. Patient adherence to drugs also could not be assessed. Fourth, some patients labeled as having RH in our study had either white-coat or pseudo-resistant hypertension, and were thus misclassified. Conversely, we likely misclassified some patients with uncontrolled hypertension on fewer than 3 medications who would remain uncontrolled on ≥3 medications as non-RH patients. Pierdomenico SD et al. have showed that patients with pseudo-resistant hypertension are at lower risk than those with true RH, and those with masked hypertension are at higher risk than those with responder hypertension [27]. Our study design may therefore underestimate the hazards of RH. Finally, the present study did not account for optimal dosing of each medication. However, medication use in the present study represents real-world management choices.

## Conclusions

Our study showed that patients with RH were associated with higher risks for cardiovascular events than those with non-RH. The elevated risks mainly contribute to increasing stroke events, especially ischemic stroke. Combining the clinical diagnosis of RH with the analysis of patient characteristics (gender and age) allows better risk stratification.
